# Phospholipase C beta 4 in mouse hepatocytes: Rhythmic expression and cellular distribution

**DOI:** 10.1186/1476-5926-7-8

**Published:** 2008-10-28

**Authors:** Brittany M Klein, Jane B Andrews, Barbra A Bannan, Ashley E Nazario-Toole, Travis C Jenkins, Kimberly D Christensen, Sorinel A Oprisan, Elizabeth L Meyer-Bernstein

**Affiliations:** 1 Department of Biology and Program in Neuroscience, College of Charleston, Charleston, SC 29424, USA; 2 Department of Physics and Astronomy, College of Charleston, Charleston, SC 29424, USA

## Abstract

**Background:**

Circadian regulated physiological processes have been well documented in the mammalian liver. Phospholipases are important mediators of both cytoplasmic and nuclear signaling mechanisms in hepatocytes, and despite a potentially critical role for these enzymes in regulating the temporal aspect of hepatic physiology, their involvement in the circadian liver clock has not been the subject of much investigation. The phospholipase C β4 (PLCβ4) enzyme is of particular interest as it has been linked to circadian clock function. In general, there is no knowledge of the role of the PLCβ4 isozyme in mammalian hepatocytes as this is the first report of its expression in the mammalian liver.

**Results:**

We found that in the liver of mice housed on a light:dark cycle, PLCβ4 protein underwent a significant circadian rhythm with a peak occurring during the early night. In constant darkness, the protein rhythm was more robust and peaked around dusk. We also observed a significant oscillation in *plcβ4 *gene expression in the livers of mice housed in both photoperiodic and constant dark conditions. The cellular distribution of the protein in hepatocytes varied over the course of the circadian day with PLCβ4 primarily cytoplasmic around dusk and nuclear at dawn.

**Conclusion:**

Our results indicate that PLCβ4 gene and protein expression is regulated by a circadian clock in the mouse liver and is not dependent on the external photoperiod. A light-independent daily translocation of PLCβ4 implies that it may play a key role in nuclear signaling in hepatocytes and serve as a daily temporal cue for physiological processes in the liver.

## Background

The phospholipase C (PLC) enzyme family participates extensively in intracellular signaling processes by catalyzing the breakdown of phosphotidylinositol bis-phosphate to generate the second messengers, inositol trisphosphate (IP_3_) and diacylglycerol (DAG) [1]. In turn, these signaling molecules regulate the release of intracellular calcium stores and promote the activation of protein kinase C [2]. In one PLC subfamily, the β class (β1, β2, β3, β4), the enzyme is often activated by the binding of various chemical substrates to their associated G-protein coupled receptors [1]. While the tissue localization of these various PLCβ isoforms can be quite extensive, there are distinct differences between their distributions [1]. For example, in liver membranes, the presence of all four PLCβ isoforms has been examined, and only PLCβ1, 2 and 3 have shown to be present this tissue [3]. As far as we are aware, there has been no study to date that has provided evidence for the expression of PLCβ4 in liver tissue as total extracts have not been previously examined.

PLCβ enzymes can be associated with many different G-protein coupled receptors, thus, it is not surprising that they have been linked to numerous physiological processes in hepatocytes. Their role in cellular responses to epidermal growth factor, angiotensin II, vasopressin or bradykinin binding on various liver processes including cell division, mitogenesis, nuclear permeability, and hypertensive responses in the cell has been the topic of much investigation [4-6]. In addition to their ability to respond to incoming information at the cell membrane, members of the PLCβ class, have also been implicated in nuclear signaling [7-10]. The PLCβ1 isoform has been most extensively studied in this context where it appears to play a role in liver regeneration and proliferation [9,11-18].

One emerging area of interest is the possible role of phopholipase C in the mammalian circadian system. Circadian rhythms are initiated by the suprachiasmatic nucleus (SCN) in the hypothalamus of the mammalian brain [19]. The SCN acts as the master pacemaker, generating and maintaining 24-hour cycles of behavior and physiology [19]. The PLCβ4 isoform is of particular relevance as it has been localized to and connected with the function of the SCN [20,21]. PLCβ4 knockout mice display an aberrant circadian phenotype and the enzyme has been implicated in various signaling pathways in SCN cells [22-24]. Moreover, we have recently shown that the levels of PLCβ4 protein in the SCN undergo a 24 hour rhythm in abundance and propose that this oscillation serves to gate incoming information to a particular time of the day [21].

In addition to the SCN, self-sustaining clocks are found in many tissues throughout the body [25]. These peripheral clocks are believed to regulate local oscillations in physiology and behavior. In the liver, the circadian regulation is quite complex [26-28]. The liver clock regulates oscillations in gene expression, particularly those that are critical to metabolic processes [29,30]. Clock disruption appears to contribute to metabolic syndrome [31-34] as well as nonalcoholic fatty liver disease [35], indicating that the timing of these processes is necessary for optimal liver function. PLC activity can be loosely linked to many rhythmic processes in the liver, but its exact involvement with regards to their temporal regulation has not been investigated.

We hypothesized, based on its potential link with the circadian system and its known role in signal transduction that PLCβ4 may be involved in temporal signaling in the liver. To investigate this, we first sought to establish whether or not PLCβ4 is expressed in mouse liver tissue. We then determined whether there is a temporal regulation in its abundance and distribution. These data will provide critical insight into the daily regulation of second messenger systems that may underlie important circadian variation in physiological processes of the liver such as cell proliferation and metabolism.

## Results

### Diurnal and circadian variation in PLCβ4 protein

Expression of PLCβ4 protein in total liver extracts was examined using Western blot analysis.

**Figure 1:**
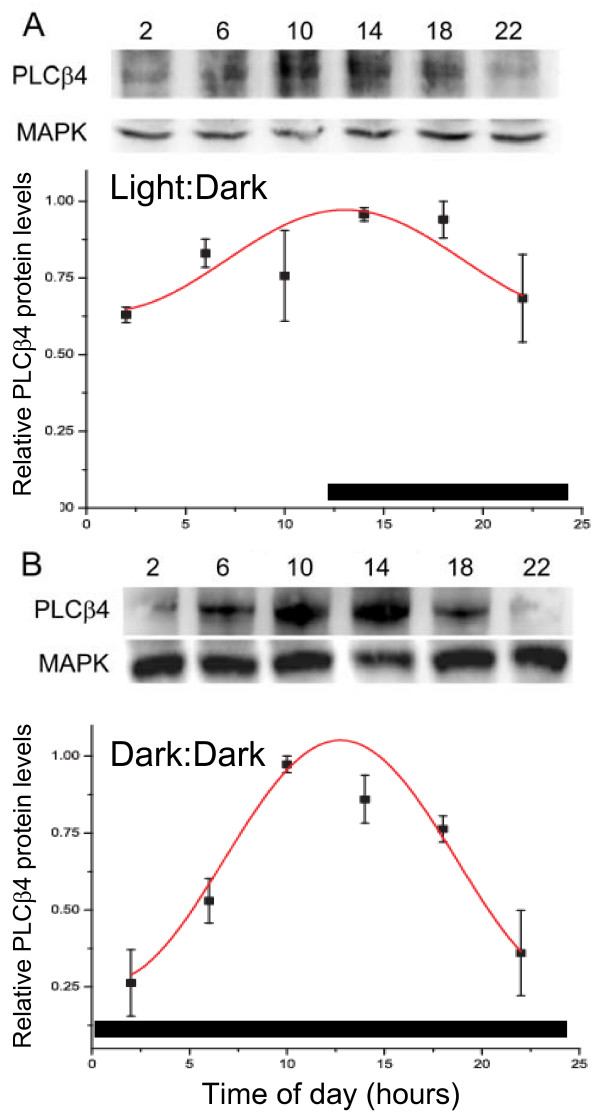
**Daily oscillation of PLCβ4 protein in the liver of mice housed in (A) a 12 hr light:12 hr dark photoperiod (Light:Dark) or in (B) constant darkness (Dark:Dark)**. Representative Western blots probed with anti-PLCβ4 antibody are shown. Each lane was loaded with 75 μg of total protein from a single mouse sacrificed at the specified time point denoted at the top of each lane. Blots were stripped and re-probed for mitogen activating peptide kinase (MAPK) to control for loading error. PLCβ4 relative optical density values were normalized to those of MAPK for each lane and then to the peak time-point for each blot. Normalized data represent mean ± SE of 3 mice at each time point. Cosinor-fitted curves have been drawn. The black bars at the bottom of each graph represent the period of darkness.

Quantification of protein in both LD and DD housed mice revealed a rhythmic oscillation (Figure 1). Statistical analysis showed a significant oscillation as a function of a 24-hr cycle in both lighting conditions. In LD, the levels of PLCβ4 protein peaked during the night at ZT10-ZT18, and were relatively low at ZT2 and 22 (Figure 1A) (ZT14, ZT18 vs. ZT2, *p *< 0.05; ZT14 vs. ZT22, *p *= 0.05). We detected a similar oscillation of PLCβ4 protein in the livers of mice housed in DD conditions (Figure 1B). Here, the protein peaked around dusk, with low levels of expression in the early morning and late night (CT 10 vs. CT 2, 6, 22, *p *< 0.0001, 0.005, 0.0005, respectively; CT14, CT18 vs. CT2, CT22, *p *< 0.015). According to the cosinor analysis, the timing of the acrophase was similar in mice housed in LD (ZT13 +/- 0.8) and DD (CT13 +/- 0.3). However, the amplitude of the DD (0.4 +/- 0.04) oscillation was much twice as robust as what was observed in the livers of the LD (0.2 +/- 0.03) mice.

### Oscillation of plcβ4 mRNA expression

**Figure 2:**
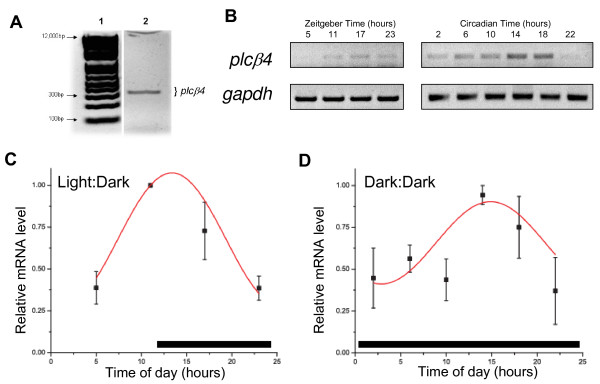
**Expression of *plcβ4 *in mouse liver tissue**. (A) PCR results using *plcb4 *set A primers and template cDNA obtained from RT-PCR of mouse liver total RNA. Lane 1: 1 kB DNA Ladder Plus (Invitrogen). Lane 2: 50 μl PCR reaction indicating *plcβ4 *product (326 bp). (B) Representative gels of *plcβ4 *gene and *gapdh *expression over the course of the day in mice housed in LD (left) and DD (right). Each lane represents data from an individual mouse sacrificed at the time of day indicated above each lane. Relative amounts of *plcβ4 *gene expression across the 24-hour day of mice housed in Light:Dark (C) or Dark:Dark (D). Normalized data represent mean ± SE of 3 mice at each time point. Cosinor-fitted curves have been drawn. The black bars at the bottom of each graph represent the period of darkness.

At the outset, we needed to confirm the presence of mRNA expression in the liver. Our initial PCR analysis demonstrates that *plcβ4 *is clearly expressed in mouse total liver extracts. A single band, approximately 300 bp in length was observed on the gel which was the expected length based on primer selection (Figure 2A). Sequencing confirmed that the band corresponds to the appropriate region of *plcβ4 *DNA. Identical results were obtained from multiple reactions using both primer sets.

In order to determine if an oscillation in *plcβ4 *gene expression underlies the rhythm in protein expression, we analyzed *plcβ4 *mRNA levels over the course of the day in the livers of mice housed in either LD or DD conditions using semi-quantitative PCR. In accordance with the protein data, the quantification of the *plcβ4 *gene expression revealed a rhythmic oscillation in the liver of mice housed in both LD and DD (Figure 2B–D). Statistical analysis showed that the mRNA displayed significant rhythmic oscillations as a function of a 24-hour cycle under both lighting conditions. In both conditions, the mRNA expression peaks around dusk and is low in the early morning and late night hours. Specifically, the *plcβ4 *mRNA is highest at dusk (ZT11) and is low in the late night (ZT11 vs. ZT23, *p *< 0.005) and early-mid day (ZT11 vs. ZT5, *p *< 0.005). In DD mice, *plcβ4 *mRNA is lowest during the early morning (CT2, *p *< 0.04, as compared to CT14) and late night (CT22, *p *< 0.02, as compared to CT14). Cosinor analysis indicates the peaks to fall at ZT13 +/- 0.8 and CT15 +/- 1.1 hours, respectively.

### Temporal translocation of PLCβ4 protein

**Figure 3:**
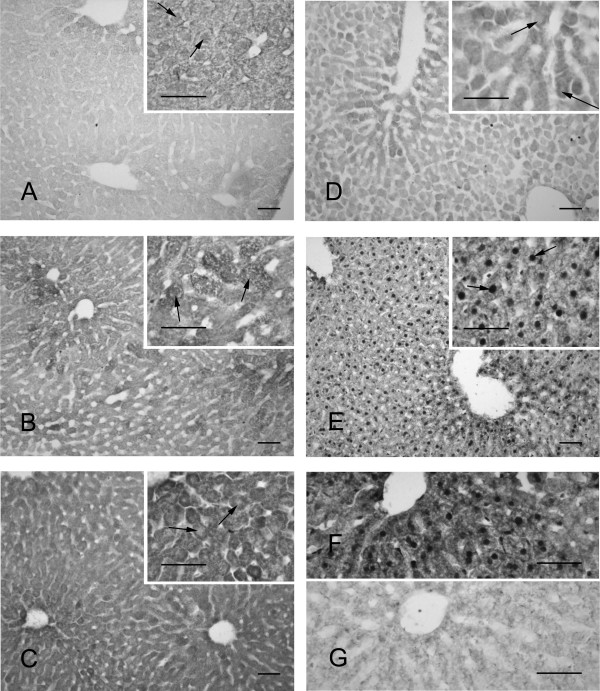
**Immunohistochemical staining for PLCβ4 in mouse hepatocytes**. In constant darkness, PLCβ4 protein fluctuated in abundance and cellular distribution throughout the circadian day. Insets: Note the change in the presence of nuclear expression over the course of the day indicated by the arrows. PLCβ4 is found in the nucleus and the cytoplasm at CT 2 (A) and transitions through CT 6 (B) to the solely cytoplasmic concentration that is noted at CT 10 (C) and 14 (D). PLCβ4 then moves back out of the cytoplasm in to the nucleus, with very high nuclear abundance prominent at CT 23 (E). Sections of liver tissue from a mouse sacrificed at CT18 incubated with rabbit anti-phospholipase C β4 primary antibody (Santa Cruz Biotechnology) (F) as compared to when the antibody is pre-absorbed with control peptide (Santa Cruz Biotechnology) prior to incubation with the tissue (G). Images are representative of 3–6 mice per time point. Scale bar = 30 μm.

Upon initial evaluation of PLCβ4 in mouse hepatocytes, we found the protein to be distributed relatively homogeneously throughout the tissue (Figure 3). In some sections, the staining appears to be more intense around the central vein, but this was not consistent. There was a marked rhythm of intracellular PLCβ4 distribution across the circadian day (Figure 3). The most obvious differences were observed between the tissue taken during the late day or early night (CT10-14) and that taken during the late night (CT23). Between CT10-14, when the total levels of PLCβ4 protein are increasing, the enzyme was primarily restricted to the cytoplasm and the nuclei were essentially devoid of PLCβ4-IR. However, during the late night, PLCβ4 was found restricted to the hepatocyte nuclei with very little IR in the cytoplasm. At the other time points sampled, the protein appeared to be in transition and was found to varying degrees in both the cytoplasm and the nucleus. These observations were consistently found in all liver tissues examined (3–6 per time point) and independent of the particular lobe that was sampled. In the control experiment where we pre-absorbed the antibody with peptide, the tissue was essentially devoid of staining (Figure 3).

## Discussion

The present study is the first to demonstrate that PLCβ4 is present in mouse liver total extracts. We found its mRNA and protein expression to be temporally regulated as well as its cellular distribution. The fact that the presence of the PLCβ4 isoform has been previously overlooked in the liver is not surprising based on its temporal expression. The low levels of the protein during the daytime would make it difficult to detect. Moreover, previous studies have restricted their analysis to membrane extracts [3], which may not be the primary location of PLCβ4 action based on the current findings.

In the present manuscript, we show that PLCβ4 protein levels undergo a robust rhythm in the mouse liver peaking during the night time hours. This temporal expression is qualitatively similar to what we previously described in the SCN [21]. A significant oscillation was observed in the livers of mice housed on both an LD cycle as well as a DD photoperiod indicating the rhythm is not being driven by an external lighting cycle. In the SCN, the rhythm in PLCβ4 was only observed in the LD mice [21]. This suggests that the enzyme likely serves different functions in these tissues and that the mechanism regulating its abundance may not be the same. That does not mean to suggest that a photic stimulus does not affect PLCβ4 protein. The observation that the protein oscillation was more robust in the absence of a light stimulus (DD), implicates light may actually serve to dampen the PLCβ4 oscillation, perhaps by affecting its stability since the mRNA oscillations appear to be of similar amplitude. In addition to light, other stimuli, most notably food availability, can serve to synchronize oscillatory processes in the liver [36]. While our data indicate photic signals do not underlie the cyclical nature of PLCβ4 gene or protein expression, we cannot rule out the possibility that a rhythm in food intake may contribute to the rhythm.

Like many mammalian peripheral tissues, hepatocytes house individual circadian clocks that serve to regulate the timing of many of its functions. The peripheral clocks can sustain 24-hr rhythms *in vitro*, but the "master clock" in the SCN is required to synchronize the intra and inter organ oscillations [37]. The exact mechanism by which the SCN controls peripheral clock machinery is not known. The signal is likely to be tissue specific and for the liver, it appears to be a blood borne constituent [38]. Historically, PLC enzymes are known to link G-protein receptors to intracellular signaling pathways by generating the second messengers, DAG and IP_3 _[1]. In this capacity, one role of the rhythmic expression of PLCβ4 in the liver may be to limit the input of extracellular signals to particular times of day as has been proposed to occur in the central brain clock [21]. While it has not been investigated for this specific isoenzyme, phospholipase C activity has been shown to mediate the downstream effects of glucagons, vasopressin, platelet activating peptide, pancreastatin and phenylephrine in the liver [3,39-41] which may impinge on various aspects of liver function. Limiting the time-of-day in which these pathways can be activated by regulating the presence of PLCβ4 would allow the liver a means by which to have temporal control of physiological functions in response to various extracellular signals. One example would be metabolism which is known to be a circadian regulated process [42-44].

The nuclear expression of PLCβ4 invokes analogies with the PLCβ1 isoform, which has been previously localized to hepatocyte nuclei [18,45]. While inositol phosphates in the liver appear to be involved in the general regulation of gene expression [46], the functional role of nuclear PLCβ1 protein in the liver has been studied primarily in the context of liver regeneration [14,47,48]. In light of the present data, PLCβ enzymes may be part of a common mechanism used to regulate cellular proliferation in the liver. Although PLCβ4 isoform has not been localized previously in hepatocyte nuclei, it has been demonstrated in the nuclei of NIH-3T3 cells where its function is not known [49]. As far as we are aware, the circadian regulated translocation has only been previously observed for proteins known to be intimately involved in the circadian clock [19,50,51]. In the liver, the differential cellular distribution pattern for the PER1 is similar to what we show here for PLCβ4, with a nuclear expression occurring in the late night and cytoplasmic expression in the late day [51,52]. Once in the cytoplasm, clock proteins necessitate dimerization prior to nuclear translocation [19]. This is not likely to be the case for PLCβ4 as it contains a carboxy-terminal nuclear translocation sequence [45]. However, this raises the possibility that an additional role for PLCβ4 may be to partner with cytoplasmic proteins and shuttle them into the nucleus during particular times of day.

Gene expression of various clock components is known to be regulated by transcription factors CLOCK and BMAL1 whose expression, in turn, is regulated by REV-ERBα, PPARα and RORα [53,54]. The mechanism by which rhythm PLCβ4 levels are controlled is not known, but the similar temporal profile initially suggested to us that it may be analogous to that of known clock genes. In the present study, we report a significant oscillation in the mRNA levels of the enzyme in both LD and DD. However, unlike the known clock genes, where the protein is typically phase delayed about 6 hours relative to mRNA expression [55], PLCβ4 protein peaks with little phase delay. These data indicate that an oscillation in mRNA, underlies the rhythm we observed in protein levels, but the regulation of *plcβ4 *gene and protein abundance is likely to differ from known clock components, and may perhaps be related to the role of PLCβ4 in signal transduction. In accordance with this, using a bioinformatics approach, we have looked into the possibility that CLOCK, BMAL and PPARα transcription factors similarly regulate *plcβ4 *gene expression by binding to E-box and PPRE promoter sequences. Thus far, we have not been able to identify any relevant promoter sequences, however, additional studies will need to be undertaken to effectively address this question. In addition to transcriptional regulation, yet to be identified post-transcriptional and post-translational mechanisms may also contribute to the oscillation in PLCβ4 protein as has been shown for other oscillating liver proteins [28]. The present observation that the amplitude of the mRNA oscillations is not directly related to that of the protein level oscillations support this idea. Alternatively, the use of a semi-quantitative PCR method, while sufficient to detect rhythm acrophase and period, may not be precise enough to make predictions regarding rhythm amplitude.

As noted here for PLCβ4, a daily oscillation in the abundance and cellular distribution of the protein may serve as a daily temporal cue in hepatocytes. Clearly, the fact that the entry of PLCβ4 into the nucleus is temporally regulated by an unknown signal adds another complexity to understanding its function. The present report opens up the possibility that PLCβ4 may contribute to temporal regulation of many physiological processes in the liver including a potential role in cellular proliferation which is regulated by the clock component, BMAL [56], and only been previously investigated for other PLCβ isoforms.

## Conclusion

The present study shows that PLCβ4 is expressed in mouse liver tissue where it undergoes a circadian oscillation. The circadian clock regulates an oscillation in gene transcription which underlies the protein cycling and may also regulate unidentified post-translational modifications of PLCβ4. The intracellular localization of PLCβ4 is also temporally regulated by a circadian clock. We conclude that the oscillatory nature of PLCβ4 likely underlies temporal signaling in hepatocytes.

## Methods

### Animals

Male mice (6–8 wk old, C57B6/J, Harlan) were housed in a 12 hr light:12 hr dark photoperiod (LD) (lights off at zeitgeber time (ZT) 12) over the course of two weeks and received food and water *ad libitum*. For mRNA and protein analysis, mice housed in LD or DD were sacrificed at designated time points over the course of the day (n = 3 mice per time point). For DD mice, the lights were turned off at ZT12 and remained off for the remainder of the experiment. Mice were sacrificed on the first full day of DD. Each mouse was euthanized with CO_2 _and fresh liver tissue was removed and immediately placed on dry ice for protein or RNA extraction. Mice were then transcardially perfused with 0.9% saline followed by 4% paraformaldehyde in 0.1 M phosphate buffer. The remaining perfused liver was used for the immunohistochemical analysis. All procedures were performed in accordance with methods approved by the College of Charleston Institutional Animal Care and Use Committee.

### Protein expression studies

Total protein from liver was extracted by homogenizing tissue in extraction buffer (0.1 M sodium chloride, 0.01 M Tris pH 7.6, 0.001 EDTA, protease inhibitor cocktail) followed by centrifugation (9000 rpm, 10 min, 2×). Supernatants were collected and quantified using BioRad Dc protein assay. 75 μg of total protein from a single mouse at each time point was separated by electrophoresis. Following overnight transfer onto a nitrocellulose membrane, blots were placed in a blocking solution (5% non-fat milk, 1% bovine serum albumin in phosphate buffered saline (PBS)) for 60 minutes. Blots were washed in PBS containing 0.3% Tween-20 (PBS-T) and probed with rabbit anti-PLCβ4 primary antibody (1:200, 120 min, 25°C, Santa Cruz Biotechnology). Membranes were washed in PBS-T and incubated in horse radish peroxidase-conjugated goat anti-rabbit secondary antibody (1:1 K, 90 min, 25°C, Jackson Immunoresearch). PLCβ4-immunofluorescent bands were visualized using SuperSignal West Dura detection kit (Pierce) and digitally imaged using a Kodak Imaging System. Membranes were stripped using Restore Western Blot Stripping Buffer (Pierce) and re-probed with anti-mitogen activated peptide kinase (MAPK) (1:10 K, Sigma). The optical density of each PLCβ4 band was quantified and was normalized to the density of the corresponding MAPK band. This antibody detects un-activated MAPK, whose levels are constant throughout the day and is often utilized as a non-cycling loading control in circadian experiments [57]. For each gel, values were normalized to daily peak levels.

### mRNA expression

Fresh liver tissue was excised and homogenized with TRIzol reagent (1 ml/100 mg tissue). After centrifugation at 4°C (10 minutes, 12,000 RPM), the RNA-containing supernatant was incubated at room temperature and chloroform (0.2 ml/1 ml TRIzol) was added to induce phase separation. RNA in the aqueous phase was precipitated with 0.5 ml isopropyl alcohol. The RNA pellet was washed with 75% ethanol (1 ml/1 ml TRIzol), briefly air dried and re-suspended in nuclease free water. Total RNA was stored at -80°C until used in RT-PCR reactions.

SuperScript First Strand Synthesis System for RT-PCR (Invitrogen) was used to generate cDNA template according to the manufacturer instructions. The initial reaction mix (10 μl) consisted of 3 μg of total RNA template, 1 μl dNTPmix, and 1 μl Oligo(dT) and DEPC treated water. Subsequent PCR reactions contained 45 μl of PCR SuperMix (Invitrogen), 1 μl of forward and reverse primer, and 3 μl template cDNA. *plcβ4 *mRNA was isolated using two primer sets that amplified overlapping regions of similar size. *plcβ4 *set A forward (GCAGGTTATATCAGGGCAGTTCC) and reverse (TTGTTGGCAGTGATAATGGTTTGT) amplified a 326 bp segment (2246–2570 bp) and *plcβ4 *set B forward (AAGACGCACGCGATTGAGTTTGTA) and reverse (CCACGTATGTCCCGATCTTCTTAT) amplified a 355 bp segment (1943–2297 bp). *gapdh *mRNA was amplified using forward (GAGCGAGACCCCACTAACATCAAA) and reverse (GAGGGGCCATCCACAGTCTTCT) primers that selected for a 341 bp segment (279–619 bp). Primers were designed using the DNA Star program (Laser Gene) and NCBI published sequences (accession numbers: NM_013829, NM_008084). PCR products were verified by the capillary electrophoretic method of sequencing (Beckman Coulter CEQ 8000).

Total liver RNA from mice sacrificed across the circadian day was extracted for semi-quantitative PCR experiments similar to those previously described [58]. RT-PCR was performed on each sample followed by PCR with *plcβ4 *set A, *plcβ4 *set B, or *gapdh *primers. To validate our semi-quantitative method, we used different PCR cycles (data not shown). Based on these results, the following PCR protocol was used: PCR amplification for 35 cycles with denaturation at 95°C for 30 s, annealing at 58.9°C for 1 min, and extension at 72°C for 3 min. The *plcβ4 *gene was always amplified at the same time as the *gapdh *gene. PCR products were loaded and run on a 1% agarose gel. The relative optical density of *plcβ4 *and *gapdh *amplified products were quantified using a Kodak Imaging System. Background optical density values were subtracted and *plcβ4 *levels were normalized to *gapdh *levels at each time point over the course of the day and then normalized to the peak time of day for each gel.

### Histological studies

Perfused livers were postfixed overnight in 4% paraformaldehyde and cryoprotected in 30% sucrose in 0.1 M PBS. Liver sections (12 μm) were cut using a cryostat and mounted directly onto gelatin coated glass slides. The sections were rinsed in 0.01 M PBS before being incubated in 1.5% H_2_O_2 _in PBS (15 min). After further rinsing in PBS, non-specific binding was blocked by incubating sections in 3% normal goat serum with 0.3% Triton X-100 prior to overnight incubation (4°C) in rabbit anti-PLCβ4 (1:200, Santa Cruz Biotechnology). A pre-absorption control was performed by incubating the antibody with 5× concentration of blocking peptide (Santa Cruz Biotechnology) at 4°C for 2 hours prior to incubation with the tissue. Following rinsing with PBS, sections were incubated in biotinylated goat anti-rabbit secondary antibody (1:200, 90 min, 25°C), and then rinsed again. Sections were then incubated in an avidin-biotin complex (1:100; 90 min, 25°C, Vectastain Elite, Vector Laboratories). After rinsing, the antibody complex was visualized using Sigma Fast™ 3,3'-diaminobenzidine tetrahydrochloride with metal enhancer. Sections were rinsed again in PBS, dehydrated and coverslipped. Immunoreactivity (IR) was observed and documented using a light microscope attached to a High Resolution FireWire Digital CCD Color Microscope Camera (Q Imaging, Burnaby, BC, Canada) attached to a PC.

### Statistical analysis

Differences in between time points of mRNA and protein expression was initially analyzed using a one-way ANOVA and Fisher's LSD post-hoc analysis and followed up with a previously described cosinor fitting [59] used to determine circadian rhythmicity. The following equation was used for the cosinor analysis: M + k1*cos(2*pi*t/24) + k2*sin(2*pi*t/24). Data were fitted using a 99% confidence level. All data are shown as mean +/- SE (n = 3 mice at each time point). All *p*-values reported in the text refer to the post-hoc analysis.

## Competing interests

The authors declare that they have no competing interests.

## Authors' contributions

BMK performed the DD RNA experiments and edited the manuscript. JBA performed the histological experiments. BAB carried out the protein cycling experiments in DD. AEN-T initiated the project and performed the protein experiments in LD. TCJ participated in the design of the study and initiated the RNA experiments. KDC carried out RNA LD experiments. SAO conducted the cosinor analysis. ELM-B was primarily responsible for the conception, design and coordination of the study and wrote and finalized the manuscript. All authors have read and approved the final manuscript.

## References

[B1] Rebecchi MJ, Pentyala SN (2000). Structure, function, and control of phosphoinositide-specific phospholipase C. Physiol Rev.

[B2] Rhee SG (2001). Regulation of phosphoinositide-specific phospholipase C. Annu Rev Biochem.

[B3] Santos-Alvarez J, Sanchez-Margalet V (1998). Pancreastatin activates beta3 isoform of phospholipase C via G(alpha)11 protein stimulation in rat liver membranes. Mol Cell Endocrinol.

[B4] Johnson RM, Garrison JC (1987). Epidermal growth factor and angiotensin II stimulate formation of inositol 1,4,5- and inositol 1,3,4-trisphosphate in hepatocytes. Differential inhibition by pertussis toxin and phorbol 12-myristate 13-acetate. J Biol Chem.

[B5] O'Brien EM, Gomes DA, Sehgal S, Nathanson MH (2007). Hormonal regulation of nuclear permeability. J Biol Chem.

[B6] Ni A, Yin H, Agata J, Yang Z, Chao L, Chao J (2003). Overexpression of kinin B1 receptors induces hypertensive response to des-Arg9-bradykinin and susceptibility to inflammation. J Biol Chem.

[B7] Cocco L, Martelli AM, Barnabei O, Manzoli FA (2001). Nuclear inositol lipid signaling. Adv Enzyme Regul.

[B8] Martelli AM, Manzoli L, Faenza I, Bortul R, Billi A, Cocco L (2002). Nuclear inositol lipid signaling and its potential involvement in malignant transformation. Biochim Biophys Acta.

[B9] Albi E, Rossi G, Maraldi NM, Magni MV, Cataldi S, Solimando L, Zini N (2003). Involvement of nuclear phosphatidylinositol-dependent phospholipases C in cell cycle progression during rat liver regeneration. J Cell Physiol.

[B10] Martelli AM, Tabellini G, Borgatti P, Bortul R, Capitani S, Neri LM (2003). Nuclear lipids: new functions for old molecules?. J Cell Biochem.

[B11] Cocco L, Martelli AM, Vitale M, Falconi M, Barnabei O, Stewart Gilmour R, Manzoli FA (2002). Inositides in the nucleus: regulation of nuclear PI-PLCbeta1. Adv Enzyme Regul.

[B12] Faenza I, Matteucci A, Bavelloni A, Marmiroli S, Martelli AM, Gilmour RS, Suh PG, Manzoli L, Cocco L (2002). Nuclear PLCbeta(1) acts as a negative regulator of p45/NF-E2 expression levels in Friend erythroleukemia cells. Biochim Biophys Acta.

[B13] Xu A, Suh PG, Marmy-Conus N, Pearson RB, Seok OY, Cocco L, Gilmour RS (2001). Phosphorylation of nuclear phospholipase C beta1 by extracellular signal-regulated kinase mediates the mitogenic action of insulin-like growth factor I. Mol Cell Biol.

[B14] Faenza I, Matteucci A, Manzoli L, Billi AM, Aluigi M, Peruzzi D, Vitale M, Castorina S, Suh PG, Cocco L (2000). A role for nuclear phospholipase Cbeta 1 in cell cycle control. J Biol Chem.

[B15] Matteucci A, Faenza I, Gilmour RS, Manzoli L, Billi AM, Peruzzi D, Bavelloni A, Rhee SG, Cocco L (1998). Nuclear but not cytoplasmic phospholipase C beta 1 inhibits differentiation of erythroleukemia cells. Cancer Res.

[B16] Manzoli L, Billi AM, Rubbini S, Bavelloni A, Faenza I, Gilmour RS, Rhee SG, Cocco L (1997). Essential role for nuclear phospholipase C beta1 in insulin-like growth factor I-induced mitogenesis. Cancer Res.

[B17] Zini N, Ognibene A, Marmiroli S, Bavelloni A, Maltarello MC, Faenza I, Valmori A, Maraldi NM (1995). The intranuclear amount of phospholipase C beta 1 decreases following cell differentiation in Friend cells, whereas gamma 1 isoform is not affected. Eur J Cell Biol.

[B18] Divecha N, Rhee SG, Letcher AJ, Irvine RF (1993). Phosphoinositide signalling enzymes in rat liver nuclei: phosphoinositidase C isoform beta 1 is specifically, but not predominantly, located in the nucleus. Biochem J.

[B19] Gachon F, Nagoshi E, Brown SA, Ripperger J, Schibler U (2004). The mammalian circadian timing system: from gene expression to physiology. Chromosoma.

[B20] Ikeda M, Sugiyama T, Suzuki K, Moriya T, Shibata S, Katsuki M, Allen CN, Yoshioka T (2000). PLC beta 4-independent Ca2+ rise via muscarinic receptors in the mouse suprachiasmatic nucleus. Neuroreport.

[B21] Jenkins TC, Andrews JB, Meyer-Bernstein EL (2007). Daily oscillation of phospholipase C beta4 in the mouse suprachiasmatic nucleus. Brain Res.

[B22] Kim D, Jun KS, Lee SB, Kang NG, Min DS, Kim YH, Ryu SH, Suh PG, Shin HS (1997). Phospholipase C isozymes selectively couple to specific neurotransmitter receptors. Nature.

[B23] Park D, Lee S, Jun K, Hong YM, Kim DY, Kim YI, Shin HS (2003). Translation of clock rhythmicity into neural firing in suprachiasmatic nucleus requires mGluR-PLCbeta4 signaling. Nat Neurosci.

[B24] von Gall C, Stehle JH, Weaver DR (2002). Mammalian melatonin receptors: molecular biology and signal transduction. Cell Tissue Res.

[B25] Nishide SY, Honma S, Nakajima Y, Ikeda M, Baba K, Ohmiya Y, Honma K (2006). New reporter system for Per1 and Bmal1 expressions revealed self-sustained circadian rhythms in peripheral tissues. Genes Cells.

[B26] Albrecht U (2006). Molecular orchestration of the hepatic circadian symphony. Genome Biol.

[B27] Davidson AJ, Castanon-Cervantes O, Stephan FK (2004). Daily oscillations in liver function: diurnal vs circadian rhythmicity. Liver Int.

[B28] Reddy AB, Karp NA, Maywood ES, Sage EA, Deery M, O'Neill JS, Wong GK, Chesham J, Odell M, Lilley KS (2006). Circadian orchestration of the hepatic proteome. Curr Biol.

[B29] Kornmann B, Schaad O, Bujard H, Takahashi JS, Schibler U (2007). System-driven and oscillator-dependent circadian transcription in mice with a conditionally active liver clock. PLoS Biol.

[B30] Akhtar RA, Reddy AB, Maywood ES, Clayton JD, King VM, Smith AG, Gant TW, Hastings MH, Kyriacou CP (2002). Circadian cycling of the mouse liver transcriptome, as revealed by cDNA microarray, is driven by the suprachiasmatic nucleus. Curr Biol.

[B31] Turek FW, Joshu C, Kohsaka A, Lin E, Ivanova G, McDearmon E, Laposky A, Losee-Olson S, Easton A, Jensen DR (2005). Obesity and metabolic syndrome in circadian Clock mutant mice. Science.

[B32] Staels B (2006). When the Clock stops ticking, metabolic syndrome explodes. Nat Med.

[B33] Karlsson B, Knutsson A, Lindahl B (2001). Is there an association between shift work and having a metabolic syndrome? Results from a population based study of 27,485 people. Occup Environ Med.

[B34] Wijnen H, Young MW (2006). Interplay of circadian clocks and metabolic rhythms. Annu Rev Genet.

[B35] Sookoian S, Castano G, Gemma C, Gianotti TF, Pirola CJ (2007). Common genetic variations in CLOCK transcription factor are associated with nonalcoholic fatty liver disease. World J Gastroenterol.

[B36] Damiola F, Le Minh N, Preitner N, Kornmann B, Fleury-Olela F, Schibler U (2000). Restricted feeding uncouples circadian oscillators in peripheral tissues from the central pacemaker in the suprachiasmatic nucleus. Genes Dev.

[B37] Yoo SH, Yamazaki S, Lowrey PL, Shimomura K, Ko CH, Buhr ED, Siepka SM, Hong HK, Oh WJ, Yoo OJ (2004). PERIOD2::LUCIFERASE real-time reporting of circadian dynamics reveals persistent circadian oscillations in mouse peripheral tissues. Proc Natl Acad Sci USA.

[B38] Guo H, Brewer JM, Lehman MN, Bittman EL (2006). Suprachiasmatic regulation of circadian rhythms of gene expression in hamster peripheral organs: effects of transplanting the pacemaker. J Neurosci.

[B39] Fischer L, Haag-Diergarten S, Scharrer E, Lutz TA (2005). Leukotriene and purinergic receptors are involved in the hyperpolarizing effect of glucagon in liver cells. Biochim Biophys Acta.

[B40] Im DS, Nagano K, Katada T, Okajima F, Ui M (2005). Differential change of Ins-P3-Ca2+ signaling during culture of rat hepatocytes. Cell Signal.

[B41] Miguel BG, Calcerrada MC, Martin L, Catalan RE, Martinez AM (2001). Increase of phosphoinositide hydrolysis and diacylglycerol production by PAF in isolated rat liver nuclei. Prostaglandins Other Lipid Mediat.

[B42] Rutter J, Reick M, McKnight SL (2002). Metabolism and the control of circadian rhythms. Annu Rev Biochem.

[B43] Kennaway DJ, Owens JA, Voultsios A, Boden MJ, Varcoe TJ (2007). Metabolic homeostasis in mice with disrupted Clock gene expression in peripheral tissues. Am J Physiol Regul Integr Comp Physiol.

[B44] Noshiro M, Usui E, Kawamoto T, Kubo H, Fujimoto K, Furukawa M, Honma S, Makishima M, Honma K, Kato Y (2007). Multiple mechanisms regulate circadian expression of the gene for cholesterol 7alpha-hydroxylase (Cyp7a), a key enzyme in hepatic bile acid biosynthesis. J Biol Rhythms.

[B45] Kim CG, Park D, Rhee SG (1996). The role of carboxyl-terminal basic amino acids in Gqalpha-dependent activation, particulate association, and nuclear localization of phospholipase C-beta1. J Biol Chem.

[B46] Alcazar-Roman AR, Wente SR (2008). Inositol polyphosphates: a new frontier for regulating gene expression. Chromosoma.

[B47] Cocco L, Capitani S, Maraldi NM, Mazzotti G, Barnabei O, Rizzoli R, Gilmour RS, Wirtz KW, Rhee SG, Manzoli FA (1998). Inositides in the nucleus: taking stock of PLC beta 1. Adv Enzyme Regul.

[B48] Crljen V, Visnjic D, Banfic H (2004). Presence of different phospholipase C isoforms in the nucleus and their activation during compensatory liver growth. FEBS Lett.

[B49] Cocco L, Rubbini S, Manzoli L, Billi AM, Faenza I, Peruzzi D, Matteucci A, Artico M, Gilmour RS, Rhee SG (1999). Inositides in the nucleus: presence and characterisation of the isozymes of phospholipase beta family in NIH 3T3 cells. Biochim Biophys Acta.

[B50] Cayetanot F, Deprez J, Aujard F (2007). Calbindin D28K protein cells in a primate suprachiasmatic nucleus: localization, daily rhythm and age-related changes. Eur J Neurosci.

[B51] Yagita K, Yamaguchi S, Tamanini F, Horst GT van Der, Hoeijmakers JH, Yasui A, Loros JJ, Dunlap JC, Okamura H (2000). Dimerization and nuclear entry of mPER proteins in mammalian cells. Genes Dev.

[B52] Zylka MJ, Shearman LP, Weaver DR, Reppert SM (1998). Three period homologs in mammals: differential light responses in the suprachiasmatic circadian clock and oscillating transcripts outside of brain. Neuron.

[B53] Reddy AB, Wong GK, O'Neill J, Maywood ES, Hastings MH (2005). Circadian clocks: neural and peripheral pacemakers that impact upon the cell division cycle. Mutat Res.

[B54] Laposky AD, Bass J, Kohsaka A, Turek FW (2008). Sleep and circadian rhythms: key components in the regulation of energy metabolism. FEBS Lett.

[B55] Hastings MH, Field MD, Maywood ES, Weaver DR, Reppert SM (1999). Differential regulation of mPER1 and mTIM proteins in the mouse suprachiasmatic nuclei: new insights into a core clock mechanism. J Neurosci.

[B56] Grechez-Cassiau A, Rayet B, Guillaumond F, Teboul M, Delaunay F (2008). The circadian clock component BMAL1 is a critical regulator of p21WAF1/CIP1 expression and hepatocyte proliferation. J Biol Chem.

[B57] Obrietan K, Impey S, Storm DR (1998). Light and circadian rhythmicity regulate MAP kinase activation in the suprachiasmatic nuclei. Nat Neurosci.

[B58] Horikawa K, Minami Y, Iijima M, Akiyama M, Shibata S (2005). Rapid damping of food-entrained circadian rhythm of clock gene expression in clock-defective peripheral tissues under fasting conditions. Neuroscience.

[B59] Fahrenkrug J, Hannibal J, Georg B (2008). Diurnal rhythmicity of the canonical clock genes Per1, Per2 and Bmal1 in the rat adrenal gland is unaltered after hypophysectomy. J Neuroendocrinol.

